# Network Approaches to Substance Use and HIV/Hepatitis C Risk among Homeless Youth and Adult Women in the United States: A Review

**DOI:** 10.4236/health.2016.812119

**Published:** 2016-08-26

**Authors:** Kirk Dombrowski, Kelley Sittner, Devan Crawford, Melissa Welch-Lazoritz, Patrick Habecker, Bilal Khan

**Affiliations:** 1 Department of Sociology, University of Nebraska-Lincoln, Lincoln, USA; 2 Department of Sociology, Oklahoma State University, Stillwater, USA; 3 REACH Lab, University of Nebraska-Lincoln, Lincoln, USA

**Keywords:** Network Science, Homeless Women, Homeless Youth, Stress Process Models, Network Ecology, Racial/Ethnic Health Disparities

## Abstract

During the United States economic recession of 2008–2011, the number of homeless and unstably housed people in the United States increased considerably. Homeless adult women and unaccompanied homeless youth make up the most marginal segments of this population. Because homeless individuals are a hard to reach population, research into these marginal groups has traditionally been a challenge for researchers interested in substance abuse and mental health. Network analysis techniques and research strategies offer means for dealing with traditional challenges such as missing sampling frames, variation in definitions of homelessness and study inclusion criteria, and enumeration/population estimation procedures. This review focuses on the need for, and recent steps toward, solutions to these problems that involve network science strategies for data collection and analysis. Research from a range of fields is reviewed and organized according to a new stress process framework aimed at understanding how homeless status interacts with issues related to substance abuse and mental health. Three types of network innovation are discussed: network scale-up methods, a network ecology approach to social resources, and the integration of network variables into the proposed stress process model of homeless substance abuse and mental health. By employing network methods and integrating these methods into existing models, research on homeless and unstably housed women and unaccompanied young people can address existing research challenges and promote more effective intervention and care programs.

## 1. Introduction

During the United States economic recession of 2008–2011, poverty levels increased substantially, as did the number of “doubled-up” households, foreclosures, those on welfare and homeless adults and children [1]. In addition to economic consequences, the recession also had negative effects on people’s health and mental health, some of which have been documented in national and regional sources [1] [2]. A handful of studies have examined the extent to which the economic consequences suffered during the Great Recession led to alcohol problems. For example, according to Burgard, alcohol consumption increased as did binge drinking and alcohol dependence [1]. Little specific attention, however, has been given to the substance use and mental health needs of homeless populations and those made homeless by the economic downturn.

Homeless and unstably housed youth [HUHY] make up one of the most vulnerable populations in the U.S. They often are victims of caretaker physical and sexual abuse [3]–[5], family disorganization [3] [6], sexual exploitation [7]–[9], hunger [10] [11], and physical and sexual assault when on the streets [3] [12] [13]. Sexual behaviors, drug use, and subsistence behaviors make them among the most susceptible to sexually transmitted infections (STIs) such as HIV/AIDS [14]–[17] and hepatitis [18]–[20], most HUHY lack conventional social support and adult mentorship [21] [22]. These combined risk factors take an enormous psychological and developmental toll. Over time, conventional opportunities diminish and the consequences of street life accumulate. This “cumulative continuity” [23] can result in a lifetime of marginality characterized by antisocial behaviors [24]–[26], involvement in the criminal justice system [27]–[29], drug abuse and dependency [30] [31], economic disadvantage [32], health problems [33], and adult psychiatric disorders [13]. Although there have been numerous studies of homeless youth, there has never been a comprehensive national study of the spectrum of HUHY that comprise this complex subpopulation.

Similarly, homeless adult [HA] women remain a significantly understudied population. Perhaps because women make up a lower percentage of homeless adults, studies of HA women are relatively scarce in comparison to both general studies of HA and studies of male HA. What few studies exist of specifically female HA are characterized by small sample sizes and restricted to a single location in predominantly large coastal cities. There are few regional or longitudinal studies of HA women in the United States. Of the few studies of female HA, a significant number focus only on specific subpopulations of HA women (*i.e.*, HIV+ samples, in-treatment/clinical samples). Although HA women are one of the fastest growing segments of the homeless population in the United States [34], they remain an understudied group [35]. What findings exist suggest HA women are an extremely vulnerable population, demonstrating comparable or higher rates of substance use and mental health needs as HA men. Similar results are obtained when HA women are compared to housed women [36] [37]. The few well-designed, large sample studies that included subsamples of homeless women are now more than a decade old [38] and existing studies do not include the entire spectrum of homeless women. Research to address this problem at regional levels and across a large US region is needed to characterize this rapidly changing population.

## 2. Network Approaches to Homeless Population Estimation

Among the more important questions confronting policy makers concerned with hard-to-reach and hidden populations is the size of the population itself [39] [40]. Though fluid and often dependent on definitions of homelessness, such data are nevertheless critical to effective programing and budgeting at the national and local levels [41]–[43]. Unfortunately, as Lee *et al.* point out, hard-to-reach populations are, by definition, equally hard-to-enumerate [44] (see also Marpsat and Razafindrastsima [45]).

Understanding the size and distribution of the full spectrum of HUHY and HA women housing needs is critical. The most recent estimates of the number HUHY range from 380,000 [46] to 1,500,000 [44]. The wide range of estimates is caused by differences in the definition of homelessness: different housing statuses [sheltered and unsheltered], idiosyncratic estimation designs, differing age criteria, and/or a focus on specific identity groups [47] [48]. Moreover, many of these estimates fail to recognize that homelessness is not a steady state [49]. HUHY are constantly shifting between short-term housing, shelters, living in institutional settings [50] (e.g., foster care and serial fosterage, group homes, and post-incarceration settings), and episodes of street homelessness. The link between the housing statuses of young people [51] and differences in sexual identity [52], gender [53], employment [54], drug use [55]–[57], mental health [13], criminal justice involvement [28] [58], victimization [59], and family support [6] are well recognized. Yet each of these axes provides unique challenges to programs aimed at reducing harm, risk, and victimization among the population. Only by knowing the extent and needs of each of these groups can we hope to sufficiently address the risk of the population as a whole. This is particularly true for HIV/HepC policy, as the social conditions of HUHY present barriers to testing, awareness building, and outreach, while those involved remain at substantial risk [15] [60]–[62]. Taken singly, these problems represent significant program challenges; together they represent a crisis for an entire population.

Beyond housing status, there is consensus that HUHY tend to cluster by identity and/or subsistence strategies, [63], further fracturing service delivery and outreach. Much of the current research, however, focuses on a single identity cluster (e.g., LGBT, travelers, sexually exploited children, gangs), leaving the relationships between these clusters largely unknown. Movement between clusters and the transfer of risk behaviors from one to another are also clear, resulting in a situation best described as a “total risk network” [64] [65]. Identity specific research, in combination with small, non-representative samples (e.g., a single shelter, or clients of a single program), neglects the web of connections between risk groups. This results in a loss of critical epidemiological information and misleading estimates of the proportions and characteristics of HUHY by identity, which can exacerbate service shortages and result in less than optimum service delivery plans.

For all these reasons, reaching the full spectrum of HUHY is crucial, and delays in doing so are costly in both personal and social terms. There is plentiful evidence that the number and length of homeless episodes are central factors in risk for victimization and stress [66]. There is also evidence that the consequences for mental health, substance abuse, and physical health are amplified by time on the streets and repeated victimization [67] [68]. All of these factors are associated with elevated levels of HIV/HepC exposure, creating a feedback loop where behavioral risk is amplified at each successive episode of housing loss.

In the 1990s, “point-in-time” estimation [48] [69] and “plant/capture” correction procedures [70] were developed as advances over conventional homeless census techniques [71]–[73] and both were used in a number of US cities [47] [74] [75] (recently, see Bezerra *et al.* [76]). Yet both point-in-time and plant/capture are dependent on the existence of total census operations for their effectiveness. The use of social network techniques for population estimation from more limited samples represents a new direction in counting hard-to-enumerate groups [77]–[80].

The most notable of the new network techniques is referred to as the “network scale-up method” (NSUM). This method has been used in research on hard-to-enumerate populations, including HIV prevalence for out of treatment populations and numbers of injection drug users [81]–[84]. Though questions have been raised in comparative trials about discrepancies between NSUM and other estimation methods [85], the concept of network-based sampling has secured an important place among those who do research with hard-to-enumerate populations, including the considerable attention gained by respondent driven sampling (RDS) techniques [86]–[89]. More recently, researchers in New York have combined ideas drawn from network scale-up methods and conventional capture/recapture techniques. These techniques were used in two prior NIJ funded studies to estimate the number of commercially sexually exploited children [90] and methamphetamine users [91]. In more recent work, these techniques were expanded to include confidence intervals and error estimation [80]. Such work holds promise for use in other locations.

## 3. Peer and Network Influences on HIV Risk among Homeless and Unstably Housed Youth

As Ennett *et al.* noted more than a decade ago [92], peer networks among young people clearly demonstrate both risk-enhancing and risk-decreasing properties that influence substance abuse and unsafe sexual behavior. More recently, researchers have framed this question as the issue of prosocial versus problematic peers, and tied it directly to HIV incidence among homeless adolescents. Rice *et al.* ’s [93] study of young people in unstable housing in Los Angeles found that the number of prosocial peers had demonstrable effects on the likelihood of HIV risk over time, building on earlier work that showed the effect of these same factors on drug choice/use [94] (see also Bousman [95] for similar results for homeless youth from San Diego, and similar network findings by Kipke *et al.* [96]). Ferguson found that for highly mobile or “transient” homeless youth, being dependent on peers, as opposed to family, was closely associated with participation in street-survival behaviors, including survival sex [97] (see Tyler [98] and Um [99] for drug use and overall risk).

Elsewhere [100], some of these same data were used to show that peer network composition for homeless adolescents changed over time, away from “prosocial” peers and toward those network connections that increased HIV risk. The latter is important, as it shows a changing trajectory of HIV risk that is activated through network mechanisms, which in turn corresponds directly to time spent on the street. Indeed, it is possible that one central and often ignored mechanism for the cumulative effect of “time on the street” can be found in the changing composition of individual social networks. Evidence for this is emerging. Working in Los Angeles, Martino *et al.* [101] found that rates of risk for HIV were higher among “traveler” youth who demonstrate long-term and long-distance migration patterns. Here, differences in risk correlated with differences in social network composition, as over time HUHY moved away from “conventional individuals and institutions” and toward others like themselves [102].

Others have examined the link between drug use and sex-based risk among HUHY [103] [104]. In work by Weber *et al.* [105], sexual risk among homeless youth was found to be influenced by the combination of partnership choices and drug use (both in kind and frequency), highlighting a theme that recurs in later research: sexual risk and risk of substance abuse are closely interrelated [65] [106] [107]. These findings echo results of research by Dombrowski *et al.* for non-street-based users of methamphetamine in New York City [91], as well as for adolescents engaged in commercial sex work [90]. Here, peer and network influences on sexual risk are important considerations. Among 249 respondents, 46% of girls, 44% of boys, and 68% of transgendered youth said that they were recruited into the commercial sex economy by “friends”, rather than by potential customers, market facilitators (pimps/“boyfriends”) or family members (p. 46). The report notes:

CSEC [commercially sexually exploited children] peer groups were not only vital to youth’s entry into the market, but also to their ability to engage the market and their decision to remain in “the life”. Some of their networks were quite extensive, and over one quarter of the teens (27%) claimed to know 20 or more CSEC youth, an additional 20% of the youth said that they knew between 10 and 20 other CSEC youth…There was a widespread ethos among CSEC youth of helping each other out, even if they did not know each other very well, and this orientation extended into the market and beyond… “We recommend customers to one another and help each other out. ‘Cause we all in the same predicament, so why not…if I could look out, I’m gonna look out” (p. 59).

Homelessness played a critical role in motivating participation. The majority of study participants were living in an unstable situation: 32% of the sample for this study described their situation as “living in the street” with an additional 44% of girls saying they lived “outside a family home” [90].

With the recognition that network peers influence risk has come the realization that peer and network influences may also affect service delivery and positive social support [108] [109]. Recent work by Rice *et al.* and Chew Ng *et al.* emphasize the potential of network ties to increase shelter use [110] and reduce substance use [111]. As these authors note, social ties can be used to facilitate peer-based interventions, yet to do so, we must know more about the overall network structures that link homeless and unstably housed young people to one another [112]. This includes ties within and across identity groups, subsistence clusters, and across the full range of housing situations. Such data are rare in a single network study. Full network topologies (network sociograms that go beyond ego networks to display the full set of connections among a group) are nearly non-existent among HUHY. Nor do we know much about the connections between clusters, or the structure of the relationship between HUHY and their non-homeless peers.

To make matters worse, most studies of homeless youth have not provided adequate comparison groups among those marginally housed and those currently experiencing an episode of homelessness who may be co-participants in many of the same activities or behaviors. As noted above, the relationship between HIV/HepC risk (via drug use and commercial/exchange sex) and the range of housing statuses remains murky; while it may be well known in some places [113] [114], it is largely unknown in others.

## 4. Stress Processes among HA Women

Both stress and substance abuse/dependence disorders (SAD) are disproportionately high among homeless adult populations. Available evidence indicates that homeless adults experience significantly more stressors than other populations, including low-income housed adults [115]. In part, this is attributable to contemporaneous stressors: the fact that homelessness itself is stressful, whether due to the events of becoming homeless or to the enduring, chronic strains associated with being on the street. Yet homelessness itself is frequently the result of prior stressors, such as childhood abuse, unemployment, intimate partner violence, and past homelessness.

Homeless women encounter more stressors and strains on average than housed women. They are more likely to be victims of violence [116] and sexual assault [117], more likely to have a current and lifetime mental disorder, and to be unemployed or under employed. A large proportion of HA women may be accompanied by children, yet other HA women do not have physical custody of their children [118] [119]. Both circumstances may operate as significant sources of stress. HA women, by virtue of being homeless and being negatively regarded by the public, may experience discrimination when seeking services or in their daily lives [120]. Their gender may also place them at risk for discrimination in wage discrepancies and employment [121].

The same bi-directional causality can be seen in the area of substance use/abuse among homeless adults. In general, substance use is one response to stress [122] [123], and has also been found to exacerbate stress [124]. Among HA women, rates of substance use disorders vary between 30% and 55% [125], and rates of binge drinking and hard drug use are also quite high [126]. Chronic or ongoing stressors increase or exacerbate existing substance use problems. In Tucker *et al*.’s study [116] comparing sheltered and housed women in Los Angeles, those living in a shelter were more likely to have experienced violence, which propelled them into homelessness, and were more likely to report an increase in alcohol/drug use in response to the violence. Furthermore, the presence of mental disorder may generate additional mental health problems and stressful experiences [127] [128], and thereby affect the development or persistence of SAD.

**Figure 1:**
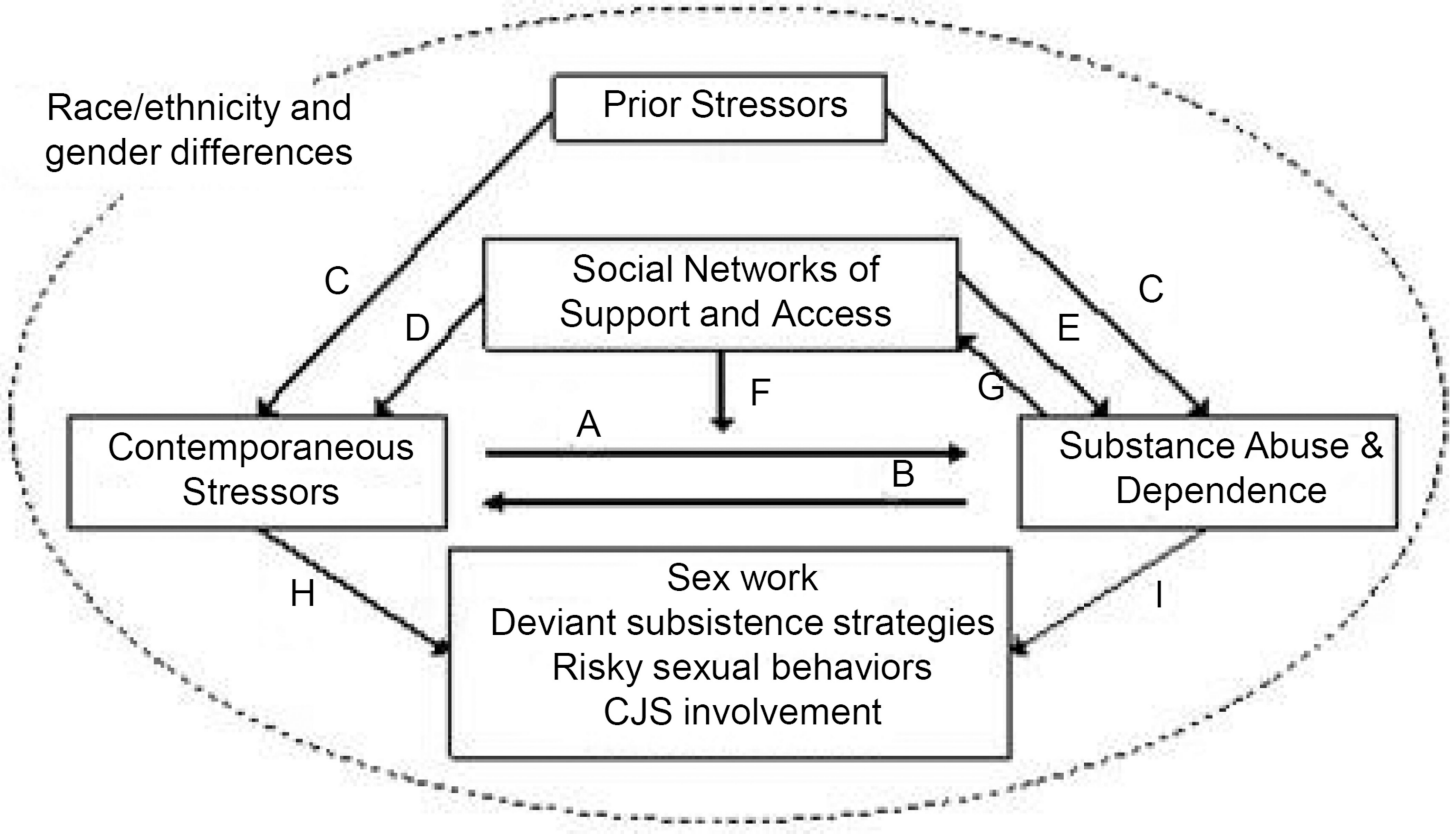
A stress process model for substance abuse and mental health among homeless populations.

These factors can be captured under a single model that combines a classic stress process model [129] [130] with Whitbeck *et al.* ’s risk amplification model [67] to consider both the bi-directional nature of the stress-substance use relationship and the buffering role of social network supports and access, as shown in Figure 1. The model links 5 core concepts in a complex model of directed and bi-directed causality. Here the major elements identified by prior researchers of homeless adults and HA women are represented as boxes: Prior Stressors, Contemporaneous Stressors, Substance Abuse and Dependence, Social Support and Exchange Networks, and a range of Homeless Outcomes that are at once contemporaneous stressors, and the behaviors/strategies most often linked to the perpetuation of homelessness (reliance on sex work survival strategies, CJS involvement, deviant subsistence strategies, and related outcomes). These 5 core concepts are related by a series of causal and bi-directional links (A through I) that predict homeless outcomes.

Thus, for example, it could be that HA women who engage in higher levels of substance use and/or have substance abuse and dependence disorders often experience more stressors (path B), whose manifestation is perpetuated by their substance abuse/dependence (path A). These together can perpetuate homeless outcomes (paths H and I; such as missed counseling appointments) that later contribute to failed efforts to exit homelessness though failures of program compliance. Examples of such simultaneous cause/effect/cause relationships are well documented in the literature: for example, being under the influence of drugs or alcohol places homeless people on the street at higher risk of being victimized [131], which in turn acts as a stressor for mental health issues, which can in turn trigger continued reliance on substance use.

This model also takes into account the role of prior stressors. Women’s experiences prior to becoming homeless, including during childhood, are often predictive of both later homelessness and SAD (path C). Stressors in childhood such as child maltreatment have been associated with risk for adult alcohol use disorders [132]. Compared to housed women, rates of childhood abuse and neglect are higher in samples of HA women [133]–[135]. It is estimated that 19% to 63% of the homeless women in prior studies had been abused or neglected as children [136] [137]. In adulthood, rates of intimate partner violence are also considerably higher among HA women than housed women [35] [116] [138]. Additionally, homeless adults are more likely to have family histories of homelessness or housing instability [139]. These prior stressors have direct effects on current stressors, contributing to their proliferation [140], as well as on current substance use problems (path C). For instance, childhood abuse is positively associated with later mental health problems among homeless youth [141]. Furthermore, prior stressors may be associated with SAD via their effects on current stressors. Among HA women, childhood abuse has been associated with chronic homelessness and substance use problems in part because of its effect on later physical abuse [30].

## 5. Network Approaches to a Stress Process Model of HA Women, as a Means to Address Racial/Ethnic Disparities

Research on the topic of homeless women and substance use/abuse is timely given recent economic changes that have increased the homeless population in suburban and rural areas. Nationally, between 2007 and 2010, the number of homeless families is estimated to have increased by 20% and the number of those families using shelters increased by 57% [142]. A recent report by the National Coalition for the Homeless indicates that the subprime mortgage crisis especially affected lower-income families and single parent families [143]. Often these families are female-headed households—showing that gender differences extend beyond individual vulnerabilities to impact homeless children as well [144]. Many of the affected households continue to live in unstable housing situations, indicating that for those on the bottom, the housing crisis is not over. For individuals having gone through the process of home loss and foreclosure, the health repercussions are just now being understood [145]–[147].

Apparent ethnic/racial differences among HA women may mask more salient social differences related to broad political economic changes, particularly in the aftermath of the social transformations that have taken place in the last several years. No current or previous study has examined how racial/ethnic outcomes may be related to differing levels of social capital and social network access in a rapidly changing rural political economy. Shelter populations in mid-size Central US cities now contain women from longstanding, historically urban African American populations, newly arrived white women displaced by changes in agricultural economies, Hispanic women who have immigrated more recently to work in the emerging rural economy, and Native American women who move between reservation and urban margins. Together, they represent a significant departure from past populations. Such personal historical differences necessarily provide different levels of social support that significantly impact both the ability to transcend homeless and deal with substance use/abuse issues.

In the Central US, the housing crisis may have both masked and accelerated ongoing rural social changes [148] [149]. Much of this transformation has been tied to changes in the meat packing industry that have attracted considerable migration from Central America [150]–[152]. Yet other deep structural changes have emerged recently as well, including differences in gendered employment [153]–[155], uneven and short-lived patterns of return migration [156] [157], differential impact of age [158] and education [159] on migration, and related general patterns of rural economic well-being [160]–[162]. One possible explanation is that these changes lie behind surface statistics documenting racial/ethnic health disparities among homeless women in the region. While little specific research has focused on this topic in the region, racial and ethnic health disparities among homeless women are well known [163]–[165].

In this view, racial and ethnic differences in homeless trajectories (and their related health outcomes) would be seen as closely related to differences in the social networks of support and influence, which themselves are products of markedly distinct roles that women of different ethnic and racial groups play in the emerging rural economy of the Central US. For white women in the region, rural economic decline has, for some, meant a descent into poverty and resulting strained family ties, internal migration, and new forms of welfare dependency. In contrast, Hispanic migrant women, many of whom lack legal documentation, can draw on little support when domestic or economic changes overwhelm personal resources. African American women in the region are primarily urban, and many are able to retain social networks despite tenuous economic status (in contrast, for example, to migrant Hispanic women). And Native American women often move in and out of social networks as conditions in home reservation communities prompt circular patterns of migration into and out of regional urban areas.

In each case, individual social networks reflect these historical differences, and are reflected in individual histories of housing instability and homelessness. If different groups, seemingly identifiable by racial or ethnic background, draw on very different networks of support, then their differential paths out of homelessness would likely be very different. To the extent that homelessness itself is a recognized vector for both substance abuse and health deterioration, the ability to tailor programs to specific needs (toward and from desired and undesired influences) represents an important step forward in our ability to meet the needs of what, on the surface, may seem a uniformly needy population.

Network considerations can be added as mediators and moderators of the new stress process model shown in Figure 1. Considerable evidence exists for this approach. Prior research has found that negative social supports and coping strategies promote drug use or drug problems, whereas positive coping and social support reduce those same problems [34]. Throughout the US, HA women have been found to lack strong positive social support systems [133] [166], which are integral to obtaining and maintaining stable housing. Social networks remain an important and understudied aspect of homeless women’s experience, that may have direct effects (path F) as well as moderating and mediating effects on stress exposure and substance use (paths D and E). First, these networks can reduce or ameliorate stressors associated with being homeless by providing important avenues of emotional and instrumental support (path D). Second, a key factor in preventing and recovering from SAD is having access to effective social support networks (path E). Conversely, some social networks may facilitate or exacerbate both the stressors associated with homelessness and SAD if antisocial, abusive, or substance using peers and family members constitute a woman’s social network. Third, social networks serve as a key moderator of the stress/SAD association (path F), whereby women who experience stressors associated with being homeless may be more likely to reduce substance use or seek treatment if they have supportive networks, compared to women with more stressors but weak social supports. Finally, social networks themselves may be influenced by the SAD of women, including the composition of the network (*i.e.*, selecting substance using network members) as well as by damaging relationships with prosocial members (path G). These networks then may be an important mediator in the substance use-to-stress association.

At stake in such a network-enabled model is the ability to better predict homeless outcomes. Being homeless often necessitates adaptive behaviors as survival mechanisms, including sex work and deviant subsistence strategies (path H). SAD may also facilitate negative behaviors associated with abuse/addiction, such as risky sexual behaviors and deviant subsistence strategies (path I). In a study of homeless young people in four cities, being unemployed and having drug problems increased the odds of engaging in risky survival strategies [97]. Furthermore, both homelessness and SAD are associated with involvement in the criminal justice system, as many of the behaviors associated with them place homeless women at risk for arrest and imprisonment. It is estimated that 20% to 52% of women who have been homeless also have a history of criminal justice system involvement [38] [167] [168].

Contextualizing this entire model is the larger question of ethnic/racial differences in homeless outcomes (dashed circle). These differences may appear in any of the relationships in the model. For instance, distributions of stress and adversity may be highest among African Americans [122], which may partially account of the disproportionately higher numbers of African American homeless women in the cities we will study. Substance abuse and dependence also varies by racial/ethnic status, with higher rates among American Indian and white women and lower rates among African American women in general population studies [169]. Negative outcomes such as CJS involvement may also be higher for racial/ethnic minority women, compared to white women. Importantly, it is very likely that access to supportive social networks will also differ across racial/ethnic groups [170], which also impacts both homelessness and SAD. In sum, race/ethnicity is an important determinate of both homeless experiences and SAD.

There are clear payoffs from defining network actors and ties more broadly: chiefly, in this model people are not only seen as sources of resources, they are also seen as sources of resource competition [171]. Given the limited resources available to HA women, individuals in the same vicinity, doing the same things, are potential competitors for the same goods and services. For example, a street corner cannot support a large number of people pan-handling in the same location. In this view, subsistence and other survival strategies, like shelter beds, exist in a finite ecology of limited resources [171]–[173]. This competition is directly related to both flows of resources and sources of stress for HA women (see, for example, the link between scarcity and stress by Ensel and Lin [174]). In short, by thinking in terms of competition, and not just resources, researchers can test specific hypotheses about behavior, substance use, and mental health changes. Future research should examine these networks at multiple instances to see how patterns of exchange and network ties evolve over time [175]–[177] and how stress and competitive pressure affects mental health outcomes and behaviors over time [178]. At a macro level, we should consider how the entire system of exchange changes over time, as well as how the connections between organizations (defined by serving the same population) evolve over time.

## 6. Network Approaches to a Stress Process Model of HA Women, as a Means to Address Racial/Ethnic Disparities

This review argues that network techniques can play an important role in understanding substance abuse and HIV/hepatitis risk among two segments of the US homeless population: homeless adult women and homeless and unstably housed youth. In the last three decades, the use of network techniques has grown significantly public health research. We have discussed evidence that supports the greater use of network data collection and analysis in three areas: 1) population enumeration of the homeless and unstably housed, especially homeless youth (using combined network scale-up and capture-recapture methods), 2) a sociometric approach to homeless adult resource use and competition (using a network ecology approach), and 3) the integration of network factors into existing stress process models aimed at understanding substance abuse and mental health interactions (using a network-enhanced stress process model shown above). Together these elements hold out promise for greater integration of existing research findings, which in turn can help intervention planners to more successfully meet the needs of a group that has grown in both size and complexity over the last decade.
